# Controlled Self-Assembly of Conjugated Polymers via a Solvent Vapor Pre-Treatment for Use in Organic Field-Effect Transistors

**DOI:** 10.3390/polym11020332

**Published:** 2019-02-14

**Authors:** Gyounglyul Jo, Jaehan Jung, Mincheol Chang

**Affiliations:** 1 Department of Polymer Engineering, Graduate School, Chonnam National University, Gwangju 61186, Korea; chogl93@naver.com; 2 Department of Materials Science and Engineering, Hongik University, Sejongsi 30016, Korea; 3 School of Polymer Science and Engineering, Chonnam National University, Gwangju 61186, Korea; 4 Alan G. MacDiarmid Energy Research Institute, Chonnam National University, Gwangju 61186, Korea

**Keywords:** poly(3-hexylthiophene), organic field-effect transistors, self-assembly, solvent vapor annealing, molecular ordering

## Abstract

A facile solution-processing strategy toward well-ordered one-dimensional nanostructures of conjugated polymers via a non-solvent vapor treatment was demonstrated, which resulted in enhancements to the charge transport characteristics of the polymers. The amount of crystalline poly(3-hexylthiophene) (P3HT) nanofibers was precisely controlled by simply varying the exposure time of solutions of P3HT solutions to non-solvent vapor. The effects of non-solvent vapor exposure on the molecular ordering and morphologies of the resultant P3HT films were systematically investigated using ultraviolet-visible (UV-vis) spectroscopy, polarized optical microscopy (POM), grazing incidence X-ray diffraction (GIXRD), and atomic force microscopy (AFM). The non-solvent vapor facilitates the π–π stacking in P3HT to minimize unfavorable interactions between the poor solvent molecules and P3HT chains. P3HT films deposited from the non-solvent vapor-treated P3HT solutions exhibited an approximately 5.6-fold improvement in charge carrier mobility as compared to that of pristine P3HT films (7.8 × 10^−2^ cm^2^ V^−1^ s^−1^ vs. 1.4 × 10^−2^ cm^2^ V^−1^ s^−1^). The robust and facile strategy presented herein would be applicable in various opto-electronics applications requiring precise control of the molecular assembly, such as organic photovoltaic cells, field-effect transistors, light-emitting diodes, and sensors.

## 1. Introduction

Semiconducting conjugated polymers (CPs) have drawn considerable attention as promising building blocks for use in opto-electronic applications including light-emitting diodes (LEDs) [1], organic photovoltaic cells (OPVs) [2,3], and organic field-effect transistors (OFETs) [4,5]. Their key advantages such as low weight, flexibility, and solution processability may support the production of low-cost, large-area devices [6,7]. The π–π stacking of CPs through p-orbital interactions renders intriguing anisotropic opto-electronic properties [8,9,10]. Poly(3-hexylthiophene) (P3HT), which consists of a conjugated aromatic backbone and solubilizing hexyl side chains, is one of the most-studied CPs because of its tailorable electrochemical properties, high hole mobility, and excellent solubility in a wide range of organic solvents [11,12,13]. However, its application in electronic devices has suffered from its relatively low charge transport capacity, which is mainly due to low crystallinity of the polymer films [14]. It is well known that amorphous regions hamper efficient charge transport between transport sites [14]. 

To overcome this challenge, many research groups have undertaken great efforts towards the development of approaches that can significantly enhance molecular ordering and by extension the charge transport of polymer films [15,16]. Representative approaches include post-deposition techniques (e.g., thermal [17] and vapor annealing [18]) and film-deposition methods (e.g., bar-coating [19] and the Langmuir–Blodgett method [20]). However, these approaches have several limitations. The post-treatment methods often alter or even ruin the favorable properties of active or substrate materials [17,18], and the film-deposition techniques suffer from problems in scaling up for the production of polymer films [19,20]. To this end, pre-solution treatment approaches using ultraviolet (UV) irradiation [21,22] or sonication [23], which can produce highly crystalline polymer thin films at large scale without requiring harmful post-treatment, have been recently reported. However, subtle variations in processing parameters, such as solution treatment time and the temperature of the sonication bath, lead to significant alternations in the polymer morphology, and thereby the scale-up of these pre-solution treatment approaches remains a challenge. Therefore, a facile and scalable approach toward P3HT with high molecular ordering is a highly demanded development. 

Herein, we report a facile, robust, and scalable strategy for the fabrication of large-area thin films with high molecular ordering via pre-treatment of P3HT solutions with a non-solvent vapor. Specifically, P3HT solutions were exposed to a non-solvent vapor (i.e., methanol), which resulted in the formation of anisotropic nanostructured P3HT aggregates via favorable π–π stacking interactions. The molecular ordering and morphology of P3HT films were correlated with solvent vapor exposure time, as indicated by the results of ultraviolet-visible (UV-vis) spectroscopy, polarized optical microscopy (POM), grazing incident X-ray diffraction (GIXRD), and atomic force microscopy (AFM). As a result of the non-solvent vapor treatment, the charge transport characteristics of the resultant films were significantly improved in comparison to those of pristine films, by approximately 5.6-fold (i.e., 7.8 × 10^−2^ cm^2^ V^−1^ s^−1^ vs. 1.4 × 10^−2^ cm^2^ V^−1^ s^−1^). Furthermore, we demonstrate the applicability of another non-solvent (i.e., *n*-hexane) in our strategy for controlling the molecular assembly of polymer chains in solution and thereby in the solidified film as well. This simple approach toward scalable fabrication of well-ordered polymer materials may be applicable in opto-electronics applications including OPVs, OFETs, OLEDs, and sensors, wherein proper molecular assembly is essential. 

## 2. Materials and Methods 

***Materials:*** Regioregular P3HT [regioregularity (RR) ≈ 96%, M_W_ ≈ 43.7 kDa, *M*_n_ ≈ 19.7 kDa] was obtained from Rieke Metals Inc. Chloroform (anhydrous) was purchased from Sigma Aldrich. Methanol (electronic grade) and *n*-hexane (extra pure grade) were obtained from OCI (Seoul, Korea) and Duksan (Ansan, Korea), respectively. All chemicals were used as received.

***Preparation of anisotropic P3HT nanoaggregates:*** P3HT solutions (3 mg/mL) were prepared by dissolving the polymer in chloroform at 55 °C for 60 min. Subsequently, a 20-mL vial containing 2 mL of the as-prepared P3HT solution was placed inside a 200-mL glass bottle filled with 15 mL of methanol for 1, 2, 3, 4, or 5 h.

***Fabrication of organic field-effect transistor:*** Bottom-gate bottom-contact OFET devices were constructed by following previously reported procedures [24]. All device substrates were cleaned with acetone, methanol, and isopropanol in succession, followed by UV-ozone cleaning for 15 m, to remove residual organic contaminants. P3HT thin films were then coated on the pre-cleaned OFET devices via spin-coating at 2000 rpm for 60 s. Finally, the devices were placed in a vacuum oven at 55 °C for 2 h to completely remove residual solvents. 

***Characterizations:*** The absorption spectra of P3HT solutions and corresponding films were recorded with a UV-vis spectrometer (Optizen 2120UV, Mecasys, Daejeon, Korea). POM images were obtained using a LEICA DM 750 P optical microscope (Leica Microsystems, Wetzlar, Germany) equipped with an iCM 3.0 digital camera (i-Solution Inc., Seongnam, Korea). Out-of-plane GIXRD data was collected at a low incident angle of 0.2° using a Panalytical X’pert Pro (Malvern Panalytical, Malvern, United Kingdom) equipped with a Cu K_α_ X-ray source operating at 45 kV and 40 mA. The morphologies of the films were imaged by AFM using a Park Systems XE-100 (Park Systems, Suwon, Korea) in tapping mode with a silicon tip (OMCL-AC160TS, Park systems). The OFET devices were tested in an argon environment using an Agilent 4155 semiconductor analyzer (Keysight technologies, Santa Rosa, CA, USA). The drain current (*I*_DS_) and gate voltage (*V*_GS_) of the OFETs were measured, and then the field-effect mobilities were calculated following a method previously reported [24]. 

## 3. Results and Discussion

**Figure 1:**
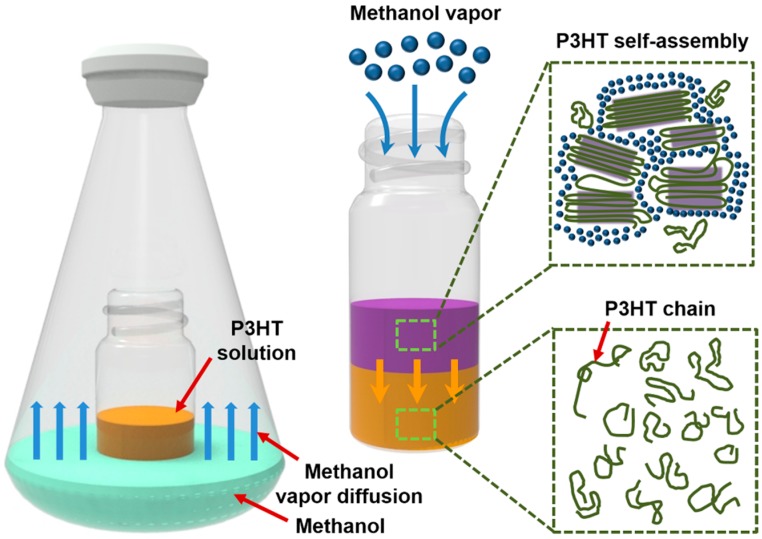
Schematic illustration of the non-solvent vapor treatment for controlling molecular self-assembly of P3HT chains.

The facile synthetic procedure for self-assembled P3HT nanofibers via solvent vapor treatment is illustrated in Figure 1. The illustration highlights the exposure of a P3HT solution to a non-solvent vapor for precisely controlling the assembly of P3HT in solution. Specifically, a vial filled with a P3HT/chloroform solution (3 mg/mL) was placed in a glass bottle containing the volatile non-solvent, methanol. As the non-solvent methanol vapors diffused into the P3HT solution in the vial, the formation of one-dimensional P3HT aggregates occurred via π−π stacking between P3HT chains, minimizing the unfavorable interactions between the polymer chains and methanol molecules [21,22].

**Figure 2:**
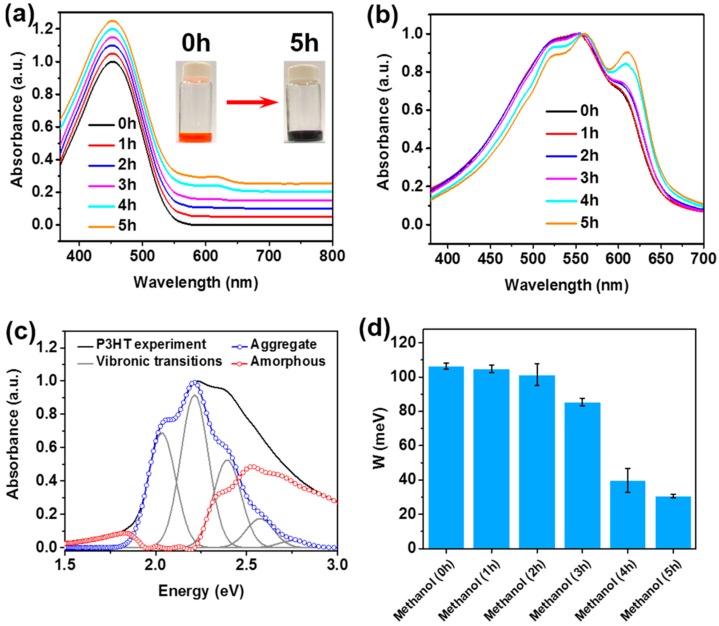
Normalized ultraviolet-visible (UV-vis) absorption spectra of P3HT (**a**) solution and (**b**) corresponding films, as functions of methanol vapor exposure time. The spectra were shifted vertically for better comparison. (**c**) Absorption spectrum of the P3HT film deposited from the solutions exposed to methanol vapor for 3 h, after being subjected to the Spano analysis using Equation (1). (**d**) Changes in exciton bandwidth (W) of P3HT films as a function of methanol vapor exposure time.

The color of the solution changed from bright orange to dark purple upon exposure to the non-solvent vapor, as shown in the inset of Figure 2a, which indicates the successful formation of well-ordered P3HT aggregates. Specifically, the color change began at the top of the solution surface, since the non-solvent molecules diffuse into the P3HT solution through the air–liquid interface. As the exposure time was increased, the color change proceeded toward the bottom of the solution (Figure S1). Figure 2a shows the UV-vis absorption spectra of P3HT solutions as a function of the non-solvent vapor exposure time, clearly indicating the formation of well-ordered P3HT aggregates via interchain interactions [21,22,25,26]. The pristine P3HT that was well-dissolved in chloroform showed only one peak at 455 nm, which was associated with the intra-chain π–π* transition of P3HT [21,22]. Upon exposure to the non-solvent vapor, however, distinct features clearly appeared in the low-energy range between ~525 (0-1 energy transition) and 650 nm (0-0 energy transition). These are associated with interchain coupling in ordered P3HT aggregates [21], supporting the conclusion that the non-solvent vapor treatment facilitates the molecular self-assembly of P3HT chains. Figure 2b shows absorption spectra of the corresponding P3HT films prepared by spin-coating. In the UV spectra of the P3HT thin films, the π–π* intraband transitions were red-shifted from the positions in the spectra of their solution state counterparts, due to the increased molecular planarity [21,22,27]. In detail, P3HT films exhibited absorption peaks at ~520 nm corresponding to the π–π* intraband transition in disordered single polymer chains, and at ~559 nm and 610 nm corresponding the interband transitions ((0-1) and (0-0), respectively) [21,28,29]. As expected, the intensities of the low-energy bands gradually increased as the time of exposure to the non-solvent vapor increased (Figure 2b), which is indicative of increased intermolecular interactions between P3HT chains [21,22]. In other words, the number of well-ordered P3HT aggregates increased with increasing exposure time. Intramolecular ordering of the polymer chains can be quantified by means of static absorption spectroscopy coupled with the Spano model [21,28]. Equation (1) explains the theoretical contribution of the well-ordered P3HT aggregates to the absorption, and was applied to the experimental spectra to attain the free exciton bandwidth (*W*) (Figure 2c and Figure S2). This value is inversely related to the degree of intramolecular ordering in a single polymer chain. 

(1) $$A\propto \sum_{m=0}\left(\frac{e^{-S}S^{m}}{m!}\right)\times {\left(1-\frac{We^{-S}}{2E_{P}}G_{m}\right)}^{2}\times \exp\left(-\frac{{\left(E-E_{0\text{-}0}-mE_{P}-1/2WS^{m}e^{-S}\right)}^{2}}{2\sigma^{2}}\right)$$

Here, *A* is the absorbance, *S* is the Huang-Rhys factor (~1.0), and *E_p_* is the vibrational energy of the C=C symmetric stretch (~0.18 eV). Methanol vapor clearly impacted the intra-molecular interactions of the resulting polymer films, as evidenced by the decrease in *W* shown in Figure 2d. Specifically, *W* was maintained at ~100 meV as the exposure time was increased from 0 to 2 h. However, a dramatic decrease in *W*, to ~31 meV, occurred upon further increasing the exposure time to 5 h. This result clearly indicates that the methanol vapor exposure led to the improved intramolecular ordering (i.e., average extent of conjugation) of the resultant P3HT films [21,30].

**Figure 3:**
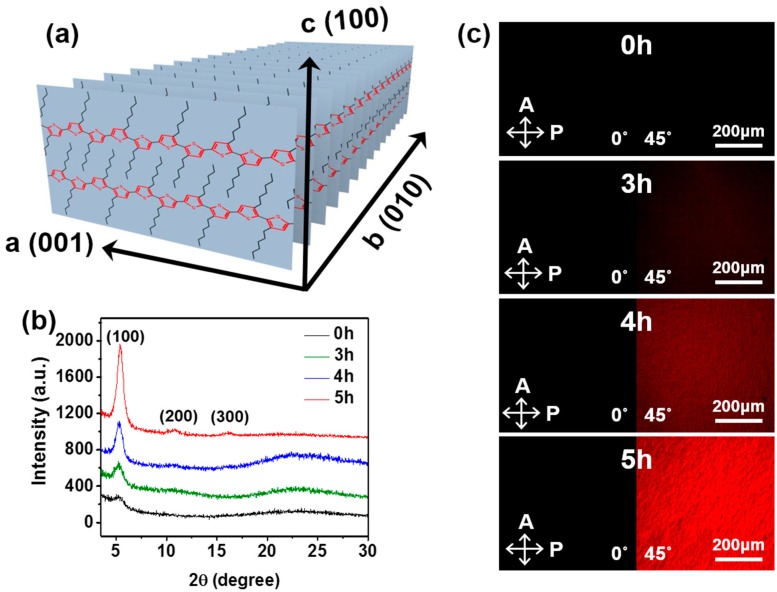
(**a**) Schematic illustration of the packing behavior of P3HT. (**b**) Grazing incidence X-ray diffraction (GIXRD) profiles and (**c**) polarized optical microscopy (POM) images of P3HT films deposited from the solutions exposed to methanol vapor for 0, 3, 4, and 5 h.

**Figure 4:**
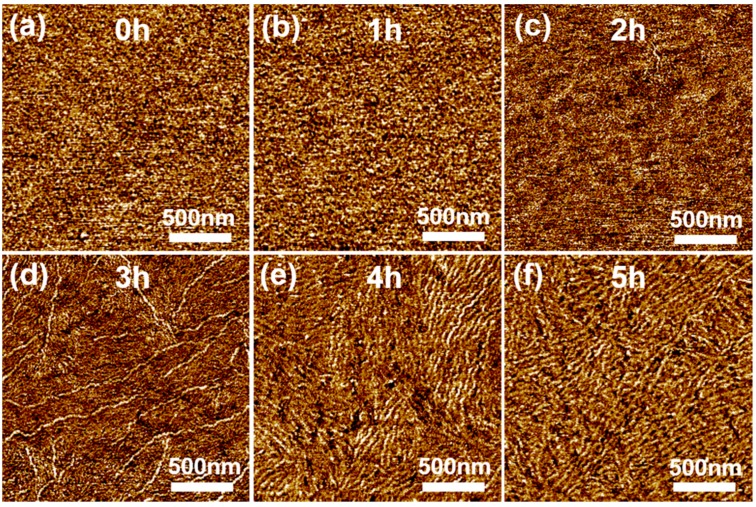
Atomic force microscopy (AFM) phase images of P3HT films prepared by spin-coating P3HT solutions exposed to methanol vapor for (**a**) 0, (**b**) 1, (**c**) 2, (**d**) 3, (**e**) 4, and (**f**) 5 h, respectively.

The intra- and inter-molecular ordering of P3HT chains are also known to correlate with film crystallinity [15,21]. Therefore, film morphology was further investigated by GIXRD measurements, the results of which are shown in Figure 3b. The intensity of the (100) peak, which is associated with the degree of lamellar packing in P3HT, increased after the non-solvent vapor treatment. This increase could be ascribed to an increase in either the size or number of crystallites, or both [24,31,32]. The 2θ value was increased from 5.28° to 5.48° after the 5 h non-solvent vapor treatment. Namely, the d-spacing of the (100) plane (Figure 3a) decreased from 1.67 to 1.61 nm. This can be explained by a change in the P3HT side chain tilt or increased interdigitation between the side chains owing to unfavorable P3HT-methanol interactions. Figure 3c represents POM images of polymer films prepared from P3HT solutions exposed to methanol vapor for 0, 3, 4, and 5 h. The birefringent texture of the films was significantly affected by variation of the non-solvent vapor exposure time. Brighter birefringent textures were observed in the films deposited from the solutions exposed to methanol vapor for longer durations. This observation is another indicator of enhanced molecular ordering [15,21]. It should be noted that the methanol vapor pre-treatment for 1 and 2 h did not result in any discernable changes to the birefringent texture of the resultant films, as compared to that of the pristine film (Figure S3). Figure 4 shows AFM images of P3HT thin films spin-coated from P3HT solutions exposed to methanol vapor for different times, from which the structures of the P3HT aggregates were revealed to be nanofibers [33,34]. The anisotropic structure of the P3HT aggregates evolved from an initial featureless structure as the exposure time increased. In solution, non-solvent vapor exposure would facilitate P3HT chain stacking via unfavorable interactions between P3HT and methanol molecules, leading to nanofibrillar aggregates. For instance, exposure of the solution to the non-solvent vapor for a short time (ca. 2 h) initiated the formation of short nanofibrils. Increased exposure time led to increases in both the number and size of nanofibers. A relatively low root-mean square (RMS) roughness (i.e., 0.56 nm) was consistently recorded for the pristine films, while the RMS roughness of the P3HT films spin-coated from the non-solvent vapor treated solutions gradually increased with increased exposure time, reaching ca. 2.4 nm. Conceivably, the increased size and amount of nanofibrillar aggregates within the films could explain the increased roughness. The results of the AFM observations are in good agreement with those of GIXRD, POM, and UV-vis spectroscopy, suggesting that the increased quantity and size of crystalline P3HT nanofibrillar structures directly afforded the increase in molecular ordering, or more specifically the crystallinity, of P3HT thin films. 

**Figure 5:**
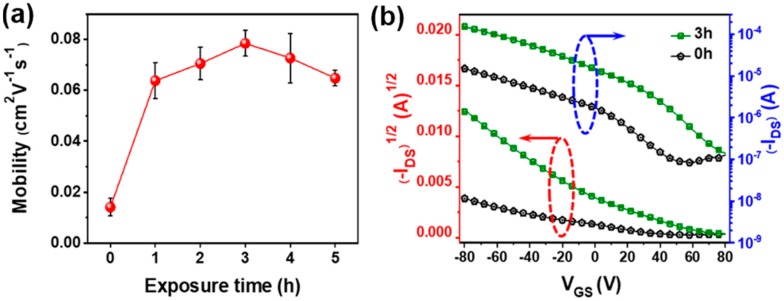
(**a**) Average field-effect mobilities of P3HT films in the saturation regime, as a function of methanol vapor exposure time. (**b**) Charge transfer characteristics of films prepared by spin-coating of P3HT solutions exposed to methanol vapor for 0 and 3 h.

To investigate how the non-solvent vapor treatment affected the charge transport behaviors of P3HT films, OFET devices with a bottom gate and bottom contact structure were fabricated by spin-coating the respective solutions onto device substrates, where the transistor channel width was 2000 μm and the length was 50 μm. Figure 5a shows the trend in field-effect mobilities of the resulting P3HT films as a function of non-solvent pre-treatment exposure time. The mobility dramatically increased by ~5.6-fold (i.e., from 1.4 × 10^−2^ to 7.8 × 10^−2^ cm^2^ V^−1^ s^−1^) as the exposure time was increased from 0 to 3 h. Interestingly, further increases in exposure time (up to 5 h) led to a decrease in the mobility to 6.5 × 10^−2^ cm^2^ V^−1^ s^−1^, despite the improved molecular ordering of the resultant films as substantiated by UV-vis spectroscopy, POM, and GIXRD analyses. This reduction is possibly a result of the excess amount of methanol favoring solute nucleation over crystal growth. As a result, the number of interfaces between crystalline regions would increase, resulting in an increase in the number of grain boundaries (i.e., the number of interfaces between crystallites) that hinder efficient charge transfer (Figure 4e,f) [7,35,36]. Figure 5b displays charge transfer curves for OFETs prepared from P3HT solutions pre-treated with methanol vapor for 0 and 3 h, and the curves obtained are characteristic of p-channel OFETs in accumulation mode. The drain current (*I*_DS_) was ~1.5 × 10^−5^ A for the pristine P3HT OFET, but ~10 times higher for the OFET prepared from the solution pre-treated for 3 h (~1.5 × 10^−4^ A). This increase is a result of the nanofibrillar P3HT aggregates formed by strong interchain interactions owing to the methanol vapor pre-treatment, which act as pathways for efficient charge transport within the film. Higher turn-on voltages (*V*_ON_) were observed in the OFET devices based on the pre-treated P3HT films than in those based on the pristine films, which was ascribed to the influences of additional doping and/or charge trapping at the interfaces between crystalline domains [5,37].

**Figure 6:**
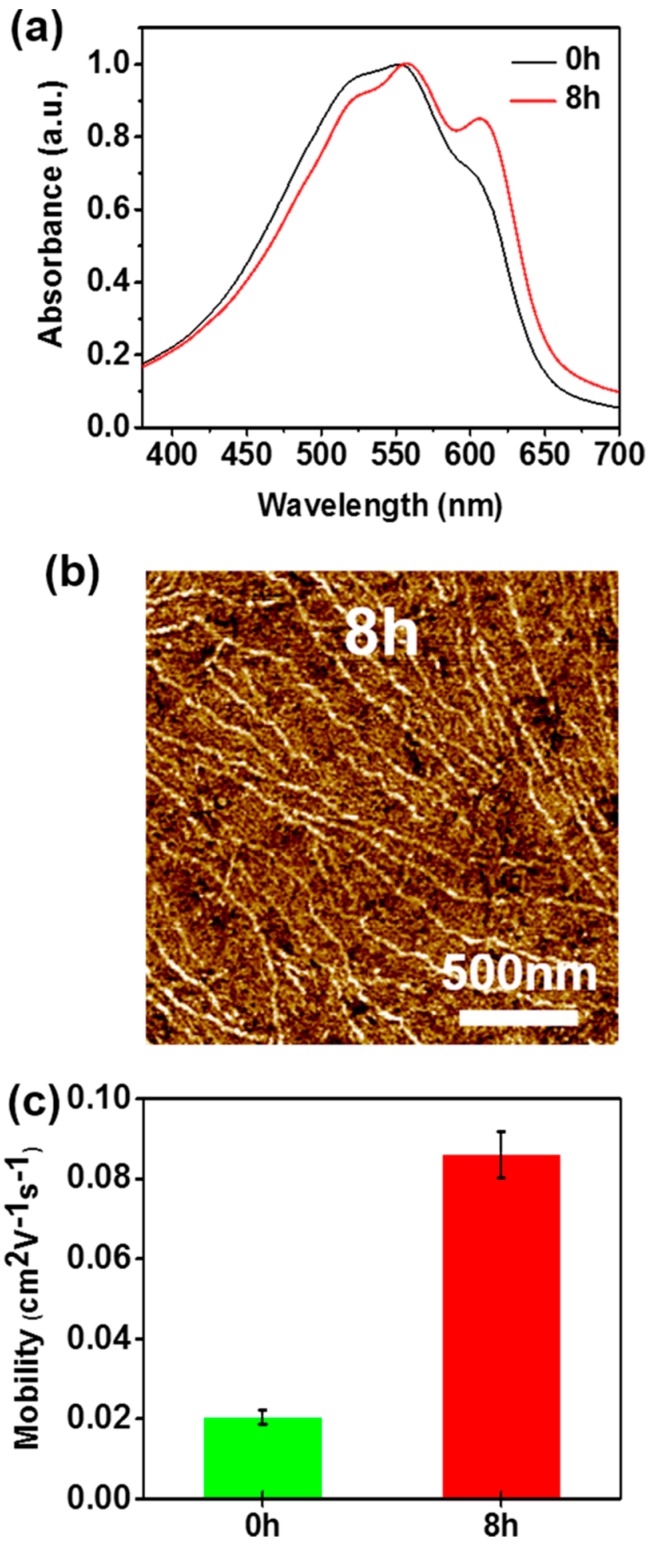
(**a**) Normalized UV-vis absorption spectra of P3HT films deposited from solutions exposed to hexane vapor for 0 and 8 h. (**b**) AFM image of the P3HT film spin-coated from the solution exposed to hexane vapor for 8 h. (**c**) Average field-effect mobilities of P3HT films spin-coated from the solutions exposed to hexane vapor for 0 and 8 h.

To investigate the general applicability of our strategy, another non-solvent, hexane, was employed in the non-solvent vapor pre-treatment. The difference in solvent polarity did not result in variations in the morphological features of P3HT films. As in the case of methanol, the treatment with hexane also led to the formation of nanofibrillar P3HT aggregates, as evidenced in the UV-vis spectra and AFM image shown in Figure 6a,b, respectively. Noticeably, the hexane-vapor-treated P3HT films exhibited lower-energy absorption features at ~559 and 610 nm which were enhanced in comparison to those of the pristine P3HT films, due to the well-ordered P3HT aggregates. Consequently, the mobility of the films prepared from the pre-treated solutions was about 4.3-fold higher than that of the films prepared from the pristine solution (i.e., 8.6 × 10^−2^ cm^2^ V^−1^ s^−1^ vs. 2.0 × 10^−2^ cm^2^ V^−1^ s^−1^). However, the non-polar solvent, hexane was found to be less preferable for the formation of crystalline P3HT aggregates compared to the polar solvent methanol. Although hexane has a higher volatility (20.1 kPa at 25 °C) than methanol (16.9 kPa at 25 °C) [38,39], the hexane vapor pre-treatment required a longer time to induce the same extent of formation of the nanofibrillar P3HT aggregates in comparison to the methanol vapor pre-treatment; this was attributed to the higher solubility of hexane in P3HT than that of methanol [40].

## 4. Conclusions

In summary, we have demonstrated the anisotropic assembly of conjugated polymers into nanofibrillar structures via a simple yet robust solution treatment strategy involving the exposure of a polymer (P3HT) solution to non-solvent vapor. Exposure of the P3HT solution to methanol vapor clearly facilitated the formation of crystalline nanofibrillar structures of P3HT via favorable π–π stacking between P3HT chains. The non-solvent vapor treatment profoundly impacted the molecular ordering, morphology, and charge transport behavior of P3HT films. Specifically, as the exposure time was increased, the degree of molecular ordering increased. The amount of crystalline P3HT nanofibers was controllable by varying the exposure time to methanol vapor. The P3HT films spin-coated from the solutions exposed to methanol vapor for 3 h exhibited mobility as a high as ~7.8 × 10^−2^ cm^2^ V^−1^ s^−1^, which is 5.6 times greater than that of the pristine P3HT films. Furthermore, we demonstrated that this approach is also applicable in the formation of nanofibrillar aggregates of crystalline conjugated polymers using another non-solvent, hexane. The strategy may also be applicable to various other polymeric materials requiring assembly into ordered structures in order to be used in a variety of commercial applications.

## Supplementary Materials

The following are available online at http://www.mdpi.com/2073-4360/11/2/332/s1: Figure S1, Photographs of P3HT/chloroform solutions in 20 mL vials after exposure to methanol vapor for 0, 1, 2, 3, 4, and 5 h; Figure S2, Absorption spectra of P3HT films spin-coated from solutions exposed to methanol vapor for 0, 1, 2, 3, 4, and 5 h; Figure S3, POM images of P3HT films spin-coated from solutions exposed to methanol vapor for 0, 1, and 2 h.
