# Supplementary material for: Controlled Self-Assembly of Conjugated Polymers via a Solvent Vapor Pre-Treatment for Use in Organic Field-Effect Transistors
Source: Polymers (Basel). 2019 Feb 14;11(2):332. doi: 10.3390/polym11020332 (PMC6419207; doi:10.3390/polym11020332)
Supplement: Supplementary file 1 [file polymers-11-00332-s001.pdf]

Supporting Information

Controlled Self-Assembly of Conjugated Polymers via a Solvent Vapor Pre-treatment for Use in Organic Field-Effect Transistors

Gyounglyul Jo [1], Jaehan Jung [2,*] and Mincheol Chang [1,3,4,*]

[1]Department of Polymer Engineering, Graduate School, Chonnam National University, Gwangju 61186, South Korea
[2]Department of Materials Science and Engineering, Hongik University, Sejongsi 30016, South Korea
[3]School of Polymer Science and Engineering, Chonnam National University, Gwangju 61186, South Korea
[4]Alan G. MacDiarmid Energy Research Institute, Chonnam National University, Gwangju 61186, South Korea
*Correspondence: jaehan@hongik.ac.kr (J.J.); mchang35@chonnam.ac.kr (M.C.); Tel.: +82-62-530-1771 (M.C.)

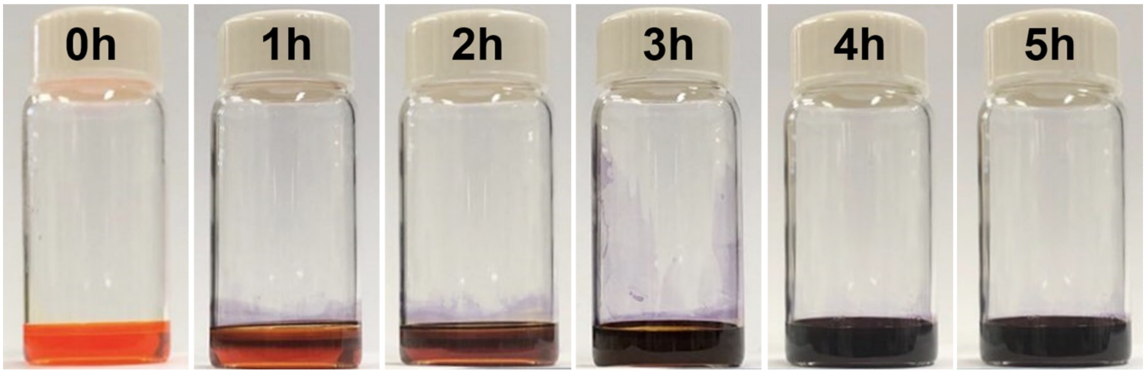

Figure S1. Photographs of P3HT/chloroform solutions in 20-mL vials after exposure to methanol vapor for 0, 1, 2, 3, 4, and 5 h.

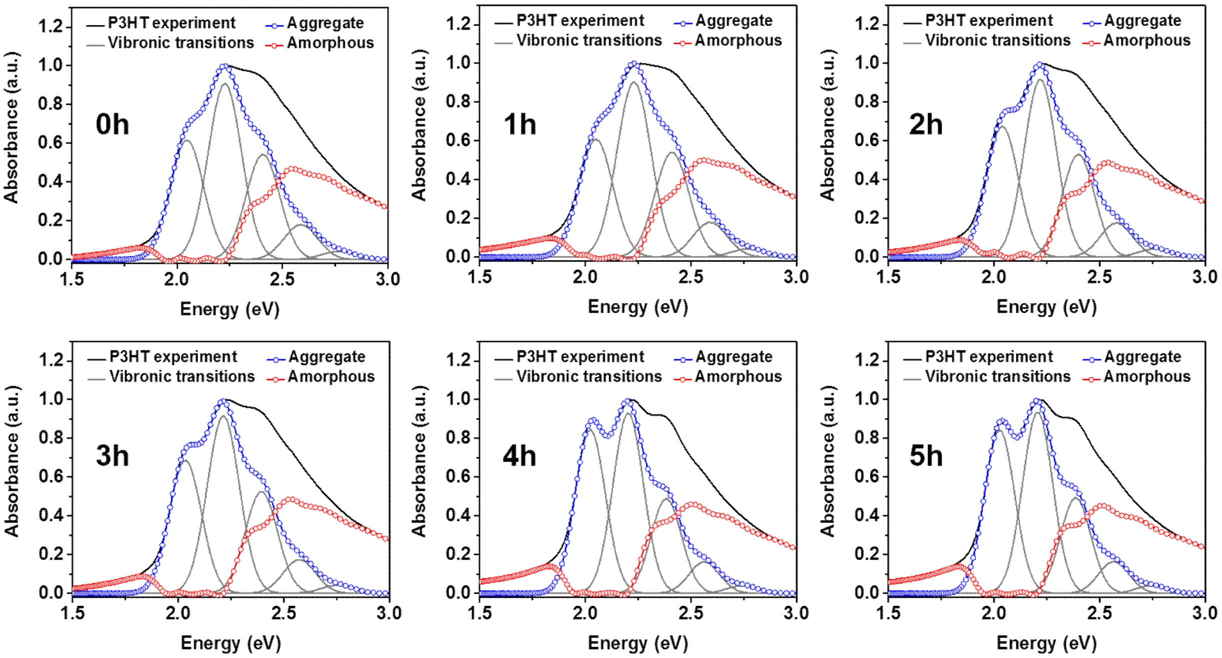

Figure S2. Absorption spectra of P3HT films spin-coated from solutions exposed to methanol vapor for 0, 1, 2, 3, 4, and 5 h. The blue and red solid lines depict the absorption spectra of P3HT aggregates and amorphous P3HT chains, respectively. The black lines represent the experimental absorption spectra.

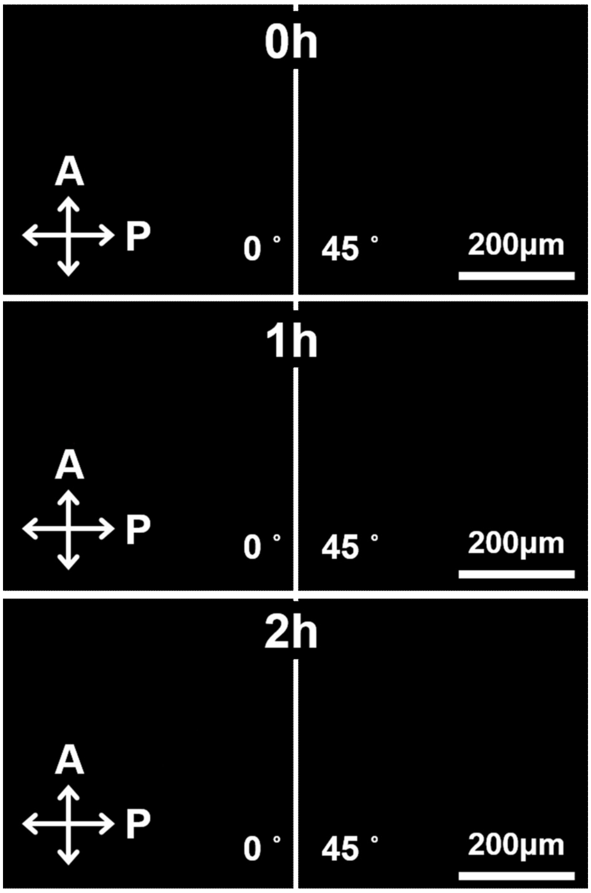

Figure S3. POM images of P3HT films spin-coated from solutions exposed to methanol vapor for 0, 1, and 2 h. **P** and **A** indicate the axes of the microscope polarizer and the light vibration plane, respectively.
